# HU of *Streptococcus pneumoniae* Is Essential for the Preservation of DNA Supercoiling

**DOI:** 10.3389/fmicb.2018.00493

**Published:** 2018-03-19

**Authors:** María-José Ferrándiz, David Carreño, Silvia Ayora, Adela G. de la Campa

**Affiliations:** ^1^Unidad de Genética Bacteriana, Centro Nacional de Microbiología, Instituto de Salud Carlos III, Madrid, Spain; ^2^Departamento de Biotecnología Microbiana, Centro Nacional de Biotecnología, Consejo Superior de Investigaciones Científicas, Madrid, Spain; ^3^Presidencia, Consejo Superior de Investigaciones Científicas, Madrid, Spain

**Keywords:** histone-like protein, HU, *Streptococcus pneumoniae*, supercoiling, nucleoid

## Abstract

The histone-like protein HU is a conserved nucleoid-associated protein that is involved in the maintenance of the bacterial chromosome architecture. It is the only known nucleoid-associated protein in *Streptococcus pneumoniae*, but it has not been studied. The pneumococcal gene encoding this protein, *hlp*, is shown herein to be essential for cell viability. Its disruption was only possible either when it was duplicated in the chromosome and its expression induced from the P*_Zn_* promoter, or when *hlp* was cloned into a plasmid under the control of the inducible P*_mal_* promoter. *In vitro* assays indicated that pneumococcal HU shows a preference for binding to supercoiled DNA rather than to linear or nicked DNA. *In vivo* experiments in which the amount of HU was manipulated showed a relationship between the amount of HU and the level of DNA supercoiling. A twofold reduction in the amount of HU triggered a 21% increase in DNA relaxation in untreated cells. However, in cells treated with novobiocin, a drug that relaxes DNA by inhibiting DNA gyrase, a 35% increase in DNA relaxation was observed, instead of the expected 20% in cells with a constitutive HU amount. Conversely, a fourfold HU increase caused only 14% of DNA relaxation in the presence of novobiocin. Taken together, these results support an essential role for HU in the maintenance of DNA supercoiling in *S. pneumoniae*.

## Introduction

*Streptococcus pneumoniae* (the pneumococcus) is the causative agent of respiratory tract infections and invasive serious illness including meningitis and bacteremia (Lynch and Zhanel, 2009). More than 1.6 million people die of pneumococcal infection every year and half of these deaths are in children aged less than 5 years according to World Health Organization estimations (World Health Organization, 2007).

As in other bacteria, the chromosome of *S. pneumoniae* is confined within the nucleoid. Chromosome compaction is achieved by the action of several factors, including DNA supercoiling, nucleoid-associated proteins (NAPs) (Pato and Banerjee, 1996) and macromolecular crowding (Wang et al., 2013). In bacteria, DNA is usually negatively supercoiled and it is mainly found in a plectonemic form (Travers and Muskhelishvili, 2007). This plectoneme formation leads to a 10% reduction in the radius of gyration of DNA, which is a measure of the volume (Luijsterburg et al., 2008). In addition, negative supercoiling eases the association of architectural proteins (de los Rios and Perona, 2007; Luijsterburg et al., 2008), which also affects the volume that a DNA molecule occupies in the cell. The DNA supercoiling balance results from the joint action of DNA topoisomerases with opposing activities (Zechiedrich et al., 2000). Topoisomerase I and topoisomerase IV relax negatively supercoiled DNA, whereas DNA gyrase introduces negative supercoiling. The alteration of this equilibrium leads to a cellular response to restore DNA superhelicity (Menzel and Gellert, 1983; Tse-Dinh, 1985; Ferrándiz et al., 2010). The large concentration of macromolecules found in the bacterial cytoplasm (>0.3 g of RNA and protein/ml in *Escherichia coli*) (Zimmerman and Trach, 1991) promotes the compaction of the DNA directly (Zimmerman, 1993) or indirectly by increasing the binding of proteins (Murphy and Zimmerman, 1995). In addition to these factors, various polycationic species neutralize DNA charges that attach DNA (de Vries, 2010).

The architectural properties of NAPs are derived from their capacity to bind DNA, wrapping it, bending the double helix or forming bridges among separate DNA segments (Luijsterburg et al., 2006; de los Rios and Perona, 2007; Luijsterburg et al., 2008). In eukaryotes the main mechanism of packaging DNA is wrapping it *via* the action of histones. Several NAPs have been described in bacteria and play an important role in the organization of chromatin (Luijsterburg et al., 2006). Members of the Lrp/AsnC family form octameric structures that wrap DNA around themselves in a right-handed superhelix to promote DNA compaction (Thaw et al., 2006; de los Rios and Perona, 2007). DNA bridging favored by NAPs leads to formation of complexes between DNA duplexes or between DNA and other architectural proteins resulting in the formation of loops. The protein archetypes of this group are H-NS and the H-NS-like proteins found in Gram-negative proteobacteria (Bertin et al., 1999; Dame et al., 2000). The proteins that bend DNA are widely distributed among prokaryotes, and the most well-known are those in the HU/IHF family (Swinger and Rice, 2004).

The architectural function of NAPs is exerted through their capacity to non-specifically bind DNA. H-NS, HU, and Fis have a preference for A+T-rich DNA, and H-NS has a greater affinity for curved DNA (reviewed by Dorman, 2014). In addition to their architectural role, NAPs have an important function in the regulation of gene expression. The preference for A+T-rich DNA allows these proteins to target horizontally acquired elements (with higher-than-average A+T content) including pathogenicity islands encoding virulence factors (reviewed by Dorman, 2014). NAPs and DNA supercoiling influences the transcription of many bacterial genes in a co-operative way, as the topological state of the DNA target is important for the binding of NAPs (Dorman, 2013). The same phenomenon occurs for H-NS and Fis, which modulate the topological response of *pel* genes (major virulence factors) in the plant pathogen *Dickeya dadantii* (Ouafa et al., 2012), and of Fis in the control of virulence genes in *Salmonella* (Croinin et al., 2006).

By bending or bridging DNA, NAPs facilitate cellular processes. HU, IHF, and Fis are involved in DNA replication (Chodavarapu et al., 2008; Kasho et al., 2014); HU participates in recombination and DNA repair (Kamashev and Rouviere-Yaniv, 2000); and H-NS, IHF, and HU act as transcriptional regulators (Dillon and Dorman, 2010; Berger et al., 2010; Kahramanoglou et al., 2011).

HU (also called HlpA, Hlp or Hup) is a small, basic, and highly conserved protein in the prokaryotic kingdom (Drlica and Rouvière-Yaniv, 1987; Azam and Ishihama, 1999) and can also be found in chloroplasts (Briat et al., 1984) and in yeast mitochondria (Caron et al., 1979). HU forms homo- or heterodimers that bind to double-stranded DNA non-specifically and with low affinity and prefers to bind intrinsically flexible DNA (Tanaka et al., 1993). A role in transcriptional regulation has been shown for this protein in *E. coli*, modulating genes that respond to anaerobiosis, acid stress, high osmolarity and SOS induction (Oberto et al., 2009). It also regulates the spatial distribution of RNA polymerase in the nucleoid, implicating a role for HU in coordinating the genomic structure and transcription (Berger et al., 2010). A role in virulence has been attributed to this protein when released into tissues during infection by *Streptococcus pyogenes* and other streptococcal species (Bergey and Stinson, 1988; Choi and Stinson, 1989, 1991; Winters et al., 1993). This virulence may be explained by the release of HU by these streptococci during the stationary phase due to the autolysis process (Stinson et al., 1998) triggering a cascade of events that induce pro-inflammatory responses that contribute to the activation of host innate immunity during bacterial infection (Liu et al., 2008a). In *S. pneumoniae*, the roles of NAPs in the chromosome architecture are unknown, and as in other streptococcal species, important proteins involved in the organization of the nucleoid, such as H-NS, IHF, or Fis, are absent. These observations suggest that HU is a key protein in the organization and compaction of the chromosome in *S. pneumoniae.* In this study, we characterized the pneumococcal HU protein (SpnHU), which is essential for the cell viability of *S. pneumoniae* due to its role in preserving DNA supercoiling.

## Materials and Methods

### Bacterial Strains, Growth and Transformation

*Streptococcus pneumoniae* R6 strain, which was used in all experiments, was grown in a casein hydrolysate-based medium (AGCH) supplemented with 0.2% yeast extract and 0.3–0.8% sucrose or 0.8% maltose as a carbon source. Transformation was performed in R6 strain with chromosomal or plasmid DNA as previously reported (Lacks et al., 1986). Transformants were selected in medium containing 1 μg/ml tetracycline for plasmids pMV158 and pMVHU (Lacks et al., 1986), and 2.5 μg/ml chloramphenicol or 250 μg/ml kanamycin for chromosomal insertions. To induce DNA relaxation, 1 μg/ml (1 × MIC) of novobiocin (NOV) was added to cultures. Strains containing plasmids pMV158, pMVHU, and pLS1 were grown in medium containing 1 μg/ml tetracycline. Growth was followed by measuring the optical density at 620 nm (OD_620__nm_) either in an UV-visible spectrophotometer (Evolution 201, Thermo Scientific) or in a microplate reader (Infinite F200, Tecan). Measurements of the two devices correlated linearly by means of the equation y = 0.2163 x + 0.1151 (y = microplate reader measure, x = spectrophotometer), with an *R*^2^ of 0.98.

### DNA Manipulation and Constructs

Chromosomal DNA and plasmids from *S. pneumoniae* were obtained as described previously (Fenoll et al., 1994). Restriction endonucleases and DNA ligase (Fermentas) were used following the supplier’s specifications. PCR was performed using 1 U of PfuI (Fermentas). Conditions for PCR were as follows: an initial cycle of 30 s denaturation at 94°C, and 30 cycles of denaturation at 94°C for 15 s, annealing at 50°C for 30 s and extension at 68°C for 1 min per kb of PCR product.

The *hlp* gene (*spr1020*) was inactivated in the chromosome by allelic replacement by homologous recombination. Three DNA fragments, obtained by PCR amplification, were digested with the appropriate restriction enzymes and ligated as follows. Two fragments upstream and downstream of the *hlp* gene of 1238 and 1151 bp were amplified with primers pairs 101829R/HU15RPAE (with the PaeI restriction site) and HU73FXBA/1021208F (with the XbaI restriction site), respectively (**Table 1**), using DNA from strain R6 as template. A third DNA fragment of 929 bp bearing the *cat* gene was amplified from plasmid pJS3 with primers CATUP1PAE and CATDOWN1XBA, which contained restriction sites for PaeI and XbaI, respectively (**Table 1**). The three fragments were digested and ligated, and the ligation product was used as a template to obtain a 3604 bp PCR product (oligonucleotide pair 101829R/1021208F), which was used to transform R6 competent cells. Transformants were selected by plating in AGCH-agar medium supplemented with 0.2% yeast extract and 0.3% sucrose and containing 2.5 μg/ml chloramphenicol. To confirm the disruption, amplification from the chromosome was performed with primers 1018131R and 1021339F (**Table 1**) flanking the replaced DNA. Primers UPHU1, CATMED, and CAT191 (**Table 1**) were used to sequence the construct.

**Table 1:** List of primers used in this work.

Primer name	Sequence (5′→ 3′)^a^	Nucleotide positions^b^
UPHU1	GCTTGGGCTATTTTGATACGT	807 to 827 of *spr1019*
HUATGSAL	gcgcggtcgacTTGGAGGAATCATTAACATG	1 to 20 of *hlp*
HUTAASAC	gcgcggagctcGACTGATTATTTAACAGCGTC	Complementary to 256 to 276 of *hlp*
HU15RPAE	gcgcgcatgcGTAGCTTCTGCTACTTTAGCG	Complementary to 24 to 44 of *hlp*
HU73FXBA	gcgcgtctagaGCAGCTTCTAAAGTACCAGCA	217 to 237 of *hlp*
HUSPH	gcgcgcatgcTAGAAAGCTTGATACAATA	Complementary to -407 to -387 of *spr1021*
HU20BIOT	BIOT-AGACTCAGCAGCAGCAGTTG	60 to 79 of *hlp*
HU61RBIOT	BIOT-CCAGTTTGTGGGTTGCGACC	Complementary to181 to 200 of *hlp*
CATUP1PAE	cgcgcgcatgcCCCATTAGTTCAACAAACG	-164 to -145 of *cat*
CATDOWN1XBA	gcgcgtctagaTATGGATCTGGAGCTGTAA	Complementary to 735 to 754 of *cat*
CATMED	CCTAACTCTCCGTCGCTATTG	Complementary to 213 to 232 of *cat*
CAT191	GTGATGGTTATCATGCAGG	575 to 593 of *cat*
101829R	TCGCCCCTCCTTCAAAGAGAT	Complementary to 89 to 109 of *spr1018*
1018131R	CCGCCAGTTGTACCTCCAGC	Complementary to 376 to 395 of *spr1018*
1021208F	CAAGAAATAACGGTCGTGGCT	646 to 666 of *spr1021*
1021339F	CTTCCCCGTCATAGCCAACAG	997 to 1017 of *spr1021*
KmR_B1_R	cgcgggatccAGGATCCATCGATACAAATTCC	Complementary to 1015 to 1037 of Kan^R^ cassette
pZK_Xb1_R	gcgctctagaCACCATAAAAAATGAACTTGG	Part of terminator sequence added
1865EXF	GGTCGTGGTGTGGATGTCGCT	703 to 723 of *spr1866*
1865EXR	ACTCCCAACCGGACCAGCAAA	Complementary to 406 to 426 of *spr1864*
GYRBRTF2	TGAAATAGTTGGAGATACGGA	483 to 503 of *gyrB*
GYRBRTR2	GAAATTTGAAGACCGCGATTT	Complementary to 612 to 632 of *gyrB*
HURTF	ATGGCAAACAAACAAGATT	1 to 19 of *hlp*
HURTR	TCACCAGCTGCAAGATAGT	Complementary to 104 to 122 of *hlp*
PARE214	AAGCGAACAGATGAAGCGATTGAG	640 to 663 of *parE*
PARE274R	TCCTTGGTGCGAACGTTATTGACA	Complementary to 822 to 845 of *parE*
TOPARTF	TCACCAAGGATGCAGTCAAAAATG	371 to 394 of *topA*
TOPARTR	GGCGAAATCGAATACCCTACCA	Complementary to 467 to 488 of *topA*
16SDNAF3	GGTGAGTAACGCGTAGGTAA	101 to 120 of 16S rDNA
16SDNAR3	ACGATCCGAAAACCTTCTTC	Complementary to 407 to 426 of 16S rDNA

*^a^ Lower case indicates bases added to the annealing sequence, and underlined sequences correspond to restriction targets. Biotinylated primers in the 5′ region are marked as BIOT-. ^b^Nucleotide numbering refers to the genes of the S. pneumoniae R6 sequence. The first nucleotide of the gene is considered nt 1.*

Plasmid pMVHU, which contains the *hlp* gene under the control of the maltose- inducible promoter P*_mal_*, was constructed as follows. First, plasmid pMV158GFP was digested with NdeI, treated with Klenow to fill in recessed 3′ ends and obtained blunted 5′ overhang, and digested again with SphI, rendering a fragment of 6186 bp without the *gfp* gene. This fragment was ligated to a 314-bp DNA obtained by amplification of R6 DNA with primers HURTF that had been previously phosphorylated and HUSPH (**Table 1**), which included a SphI restriction site, and digested with this enzyme. This same DNA fragment of 314 bp digested with SphI was also cloned into plasmid TAGZyme pQE-1 from Qiagen and, digested with PvuII and SphI to obtain six histidine (H_6_) codons fused to the 5′ end of *hlp*. The new plasmid was named pQEHU.

To clone *hlp* under the control of the Zn-inducible promoter P*_Zn_*, this copy was positioned in the chromosome of pneumococcal R6 strain at the dispensable *spr1866* locus (Martín-Galiano et al., 2014) as follows. The gene was amplified with HUATGSAL and HUTAASAC primers (**Table 1**) containing SalI and SacI restriction sites, respectively. This PCR product was digested with SalI and SacI and ligated into plasmid pZ0479 (Martín-Galiano et al., 2014). The obtained plasmid, pZ0479HU, was used as a DNA template to amplify *hlp* under P*_Zn_* together with a kanamycin resistance cassette (**Figure 2**) using the oligonucleotide pairs KmR_B1_R/ pZK_Xb1_R (**Table 1**), and attached to regions flanking the disposable *spr1866* gene (Martín-Galiano et al., 2014). This construct was introduced into the *S. pneumoniae* R6 chromosome by genetic transformation. Successful integration was checked by PCR using primers 1865EXF and 1865EXR (**Table 1**) flanking the replaced DNA. Primers HUATGSAL and HUTAASAC (**Table 1**) were used to sequence the construct.

### Southern Blot Analysis

Chromosomal DNA (3 μg) from R6, R6 P_Zn_-*hlp*, and R6 P_Zn_-*hlp*Δ*hlp* was digested with HindIII and separated by 0.8% agarose gel electrophoresis, transferred to Nylon membranes and hybridized to a biotinylated 141-bp *hlp* probe obtained by PCR amplification with 5′ biotinylated oligonucleotides HU20BIOT and HU61RBIOT (**Table 1**). Blots were developed with the Phototope-Star Detection Kit (New England Biolabs) following the manufacturer’s instructions.

### SpnHU Purification

For overexpression of *hlp* fused to H_6_ codons at its 5′ end, *E. coli* XL1 (pQEHU) was grown to an OD_600__nm_ of 0.5–0.6, and expression was induced for 2 h with 1 mM IPTG. Cells from 2 l cultures were harvested, suspended in lysis buffer (50 mM phosphate buffer pH 8.0, 300 mM NaCl, 10 mM imidazole), and chilled on ice for 1 h with 1 mg/ml lysozyme and 0.25% Triton X-100. Lysis was completed by five cycles of 20 s of sonication with an amplitude of 30% in a Sonifier B-12 (Branson Co, Danbury, CT, United States). Cell debris was pelleted by centrifugation at 20000 ×*g* for 10 min, and the resulting supernatant was filtered through a 0.45 μm Millipore Millex^®^ HA filter. This supernatant was loaded into a Ni-NTA (QIAGEN) column in an ÄKTA FPLC system following the manufacturer’s instructions. (H_6_)SpnHU was eluted with 20 ml of buffer containing a 200–350 mM gradient of imidazole. Fractions of 1 ml were collected and analyzed by SDS-12% polyacrylamide gel electrophoresis and stained with Coomassie blue. Fractions containing a protein of the expected size were dialyzed against 20 mM NaH_2_PO_4_, 150 mM NaCl, pH 7.0. To complete removal of the H_6_-tag and additional amino acids from the amino terminus, (H_6_)SpnHU was treated with the TAGZyme System (Qiagen) following the manufacturer’s instructions. This system consists of dipeptidyl aminopeptidase I (DPase I) in combination with glutamine cyclotransferase (Qcyclase) and pyroglutamyl aminopeptidase (pGAPase). The three enzymes contain a C-terminal His tag that allows their removal from the reaction solution by additional Ni-Affinity chromatography steps. The purified SpnHU was dialyzed in 10 mM TrisHCl pH 8.0, 50 mM KCl, 0.5 mM DTT, 0.5 mM EDTA and 50% glycerol and stored at -20°C. The protein concentration was measured using a colorimetric protein assay (Bio-Rad) following the manufacturer’s instructions. The concentration of SpnHU is expressed in monomeric form.

### Cross-Linking of SpnHU

SpnHU was incubated at room temperature for 30 min in 0.1% glutaraldehyde in a total volume of 10 μl containing 5 mM Tris-HCl, pH 8.0, 25 mM KCl, 25% glycerol, 0.25 mM EDTA, 0.25 mM DTT. Samples were diluted with an equal volume of Laemmli sample buffer, loaded into an SDS-polyacrylamide gel (15%), electrophoresed, and stained with Coomassie blue.

### Electrophoretic Mobility-Shift Assay (EMSA)

Plasmid pBR322 DNA (2 nM) that was supercoiled, PstI-linearized, or nicked with Nt. BstNBI (New England Biolabs) was incubated at room temperature for 20 min with different amounts of purified SpnHU in 15 μl of 10 mM Tris-HCl, pH 8.0, 50 mM KCl, 0.05% Brij 58, 0.01 mg/ml BSA, 5% glycerol, 0.01 mM EDTA and 0.01 mM DTT. Samples were loaded in a 0.5% agarose gel and electrophoresed in Tris-borate-EDTA at 18 V for 14 h at room temperature. After electrophoresis, the gels were stained with a 0.5 μg/ml ethidium bromide solution.

### Supercoiling Assays

Supercoiled plasmid pBR322 (0.18 nM) was relaxed with 5.3 nM topoisomerase I of *S. pneumoniae* obtained in our laboratory (García et al., 2011) in a final volume of 190 μl by incubation at 37°C for 30 min in 20 mM TrisHCl pH 8.0, 100 mM KCl, 10 mM MgCl_2_, 1 mM DTT, 50 μg/ml BSA. Increasing concentrations of SpnHU were added to the mix to reach a final volume of 200 μl. Reactions were incubated for 1 h at 37°C and stopped by addition of 50 mM EDTA, 1% SDS and 100 μg/ml proteinase K, followed by incubation at 37°C for 1 h. DNA was precipitated and suspended in 1 × loading buffer, electrophoresed on a 1.2% agarose gel at 18 V for 18 h, and stained with 1 μg/ml ethidium bromide.

### Two-Dimensional Agarose Gel Electrophoresis

The distribution of topoisomers of plasmid pMVHU was analyzed in neutral/neutral two-dimensional agarose gels. Plasmid DNA was obtained as previously described (Martín-Parras et al., 1998). The first dimension was run in a 0.4% (w/v) agarose (Seakem; FMC Bioproducts) gel in Tris-borate-EDTA containing 1 μg/ml of chloroquine (Sigma) at 1.5 V/cm at room temperature for 19 h. The second dimension was performed in 1% agarose gel in the same buffer containing 2 μg/ml of chloroquine at 7.5 V/cm for 7–9 h at 4°C. Chloroquine was added to both, the agarose and the running buffer. After electrophoresis, gels were subjected to Southern hybridization using as the probe a 240-bp PCR fragment obtained from pMVHU DNA with primers REPUPBIOT (5′ biotinylated) and REPDOWN (Ferrándiz et al., 2010). Chemiluminescent detection of DNA was performed with the Phototope^®^-Star kit (New England Biolabs). Images were captured in a VersaDoc MP400 system (BioRad) and analyzed with the Quantity One program. The DNA linking number (Lk) was calculated by quantifying the amount of every given topoisomer. The DNA supercoiling density (σ) was calculated with σ = ΔLk/Lk_0_. Linking number differences (ΔLk) were determined with the equation ΔLk = Lk - Lk_0_, in which Lk_0_ = N/10.5, where N is the DNA size in bp and 10.5 is the number of bp per complete turn of B-DNA, the most probable helical repeat of DNA under the used conditions.

### RNA Extraction and Real Time qRT-PCR Experiments

RNA was extracted from 2.5 to 3 × 10^10^ cells with the RNeasy kit (Qiagen). cDNAs were synthesized from 5 μg of RNA with SuperScript^™^ III Reverse Transcriptase (Invitrogen) for 1 h at 55°C. These cDNAs were subjected to quantitative qRT-PCR (Chromo 4, Bio-Rad) as described previously (Ferrándiz and de la Campa, 2014). The oligonucleotide pairs used are indicated in **Table 1**. To normalize the three independent qRT-PCR values, they were divided by those obtained by amplification of an internal fragment of 16S rDNA (**Table 1**).

### Western Blot Analysis

Rabbit polyclonal antibodies against SpnHU were obtained after three subcutaneous injections at 3-week intervals of polyacrylamide portions containing (H_6_)SpnHU. Blood was recovered and the serum was stored at -80°C. Polyclonal antibodies against LytA were kindly provided by Ernesto García (CIB, CSIC, Madrid, Spain). Whole cell lysates were obtained by centrifugation of approximately ≈ 5 × 10^10^ cells, suspended in 400 μl of phosphate buffered saline and sonicated 3 × 20 s with a Vibra Cell 75043 (Bioblock Scientific). Lysates were separated on 4–20% Mini-Protean^™^ TGX gels (Bio-Rad) and transferred to PVDF membranes. Membranes with transferred proteins were probed with anti-SpnHU (diluted 1:100) and anti-LytA (diluted 1:20000) antibodies. Super Signal West Pico chemiluminescent substrate (Thermo Scientific) was used to develop the membranes and monitored with a ChemiDoc^™^ MP system (Bio-Rad). Image analysis was performed with Image Lab^™^ software (Bio-Rad).

### Measurement of the Intracellular Amount of SpnHU

To calculate the number of SpnHU molecules per cell, Western blotting was performed using anti-SpnHU antibodies as previously described (Ali Azam et al., 1999). Briefly, quantities between 1 and 0.062 μg of purified SpnHU, and lysates of R6 cells from 5.9 × 10^7^ ± 0.71 × 10^7^ colonies-forming units (CFUs) were loaded, electrophoresed in 4–20% gels, and transferred to PVDF membranes. Blots were developed and scanned to quantify the band intensities as indicated above. The linear correlation between the amount of SpnHU and the immunostaining intensity served to interpolate the amount of protein from the cell lysate. CFUs were calculated by plating cell extracts on blood agar plates. The molecular mass of SpnHU is 9.6 kDa. Determination was performed with three independent cell extracts.

### Statistical Analysis

GraphPad Prism 7.02 was used for the statistical analysis. The SpnHU level among ΔHU (pMVHU) cells grown either with sucrose or maltose and R6 was determined by one-way analysis of variance. Supercoiling density values (σ) were determined using two-way analysis of variance with Tukey’s multiple comparisons test. The 95% confidence interval was used. Statistical significance was defined as *P* < 0.05.

## Results

### HU from *Streptococcus pneumoniae* R6 Is Highly Conserved Among Pneumococcal Strains and Streptococci of the Mitis Group

**FIGURE 1:**
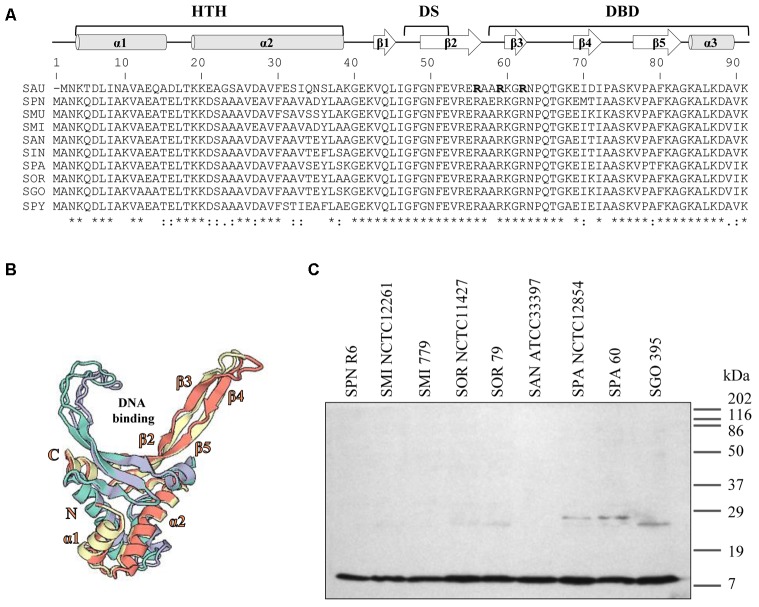
Streptococcal HU homologs are highly conserved. **(A)** Multiple sequence alignment of streptococcal HU homologs and *Staphylococcus aureus* (SAU) HU protein (SauHU). Above the alignment, secondary structural elements are shown. The secondary structure was predicted based on the position specific-scoring matrices generated by PSI-BLAST with the Pyre2 portal for protein modeling, prediction, and analysis (Kelley et al., 2015). HTH, helix-turn-helix; DS, dimerization signal; and DBD, DNA-binding domain. Residues that are crucial for DNA recognition and binding are denoted in bold face and shadowed. Below the alignment, conserved residues are identified by asterisks. SPN (*Streptococcus pneumoniae*), SMU (*Streptococcus mutans*), SMI (*Streptococcus mitis*), SAN (*Streptococcus anginosus*), SIN (*Streptococcus intermedius*), SOR (*Streptococcus oralis*), SGO (*Streptococcus gordoni*), SPA (*Streptococcus parasanguinis*), and SPY (*Streptococcus pyogenes*). **(B)** The 3D model of the SpnHU homodimer based on the crystal structures of SauHU (4qjn, homodimer subunits in green and purple) and SMU (5fbm, homodimer subunits in yellow and pink). This structure has been modeled with coverages of 98% (SAU) and 100% (SMU) of the SpnHU sequence using Swiss-Model (Biasini et al., 2014). **(C)** Western blot analysis of HU protein in different streptococcal species. Crude cell extracts (20 μg) were separated by SDS-PAGE, blotted, and incubated with an antibody directed against SpnHU protein.

Analysis of the genomic sequence of *S. pneumoniae*, revealed a single gene encoding SpnHU, suggesting that the protein is a homodimer, as in other Gram-positive bacteria (Tanaka et al., 1984; Stinson et al., 1998; Liu et al., 2008b). SpnHU from R6 shares 59 and 55% of identity with *Escherichia coli* HU subunits alpha and beta, respectively. The identity increased to 75.3 and 89% when compared with HU from Gram-positive bacteria such as *Staphylococcus aureus* (SauHU) and *Streptococcus mutans* (SmuHU), respectively. When compared with HU of streptococcal species of the mitis group, the identity increased to 95.6%. Its predicted secondary structure included three α-helices and five β-sheets following the α1-α2-β1-β2-β3-β4-β5-α3 organization (**Figure 1A**). This secondary structure is highly conserved among HU proteins and is divided in three domains: helix-turn-helix (HTH), dimerization signal (DS) and DNA-binding domain (DBD) (Christodoulou and Vorgias, 2002; Christodoulou et al., 2003). The predicted structure of the SpnHU homodimer based on SauHU and SmuHU crystal structures is shown in **Figure 1B**. This structure in SauHU and SmuHU has a V-like form that is transversally divided in two parts: an α-helical body and two protruding β-ribbon arms, which have a flexible nature and clamp DNA along the minor groove (Kim et al., 2014; O’Neil et al., 2016).

Western blot analysis using a polyclonal antibody directed against SpnHU revealed a protein with the same size as SpnHU (9.6 kDa) in the different streptococcal extracts (**Figure 1C**), highlighting the high identity among streptococcal HU proteins.

### The Biochemical Behavior of SpnHU Is Similar to That of Other HU Proteins

**FIGURE 2:**
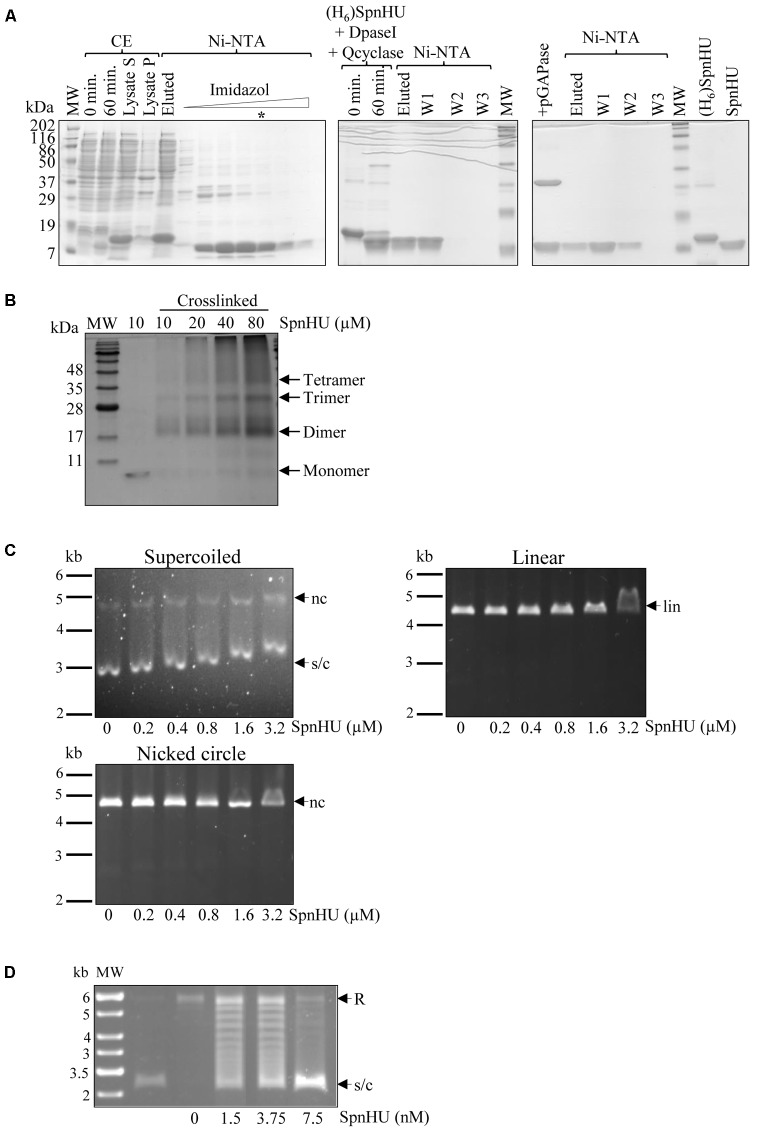
*In vitro* characterization of SpnHU activity. **(A)** Purification of recombinant [(H_6_)SpnHU] and native (SpnHU) proteins. A culture of *E. coli* XL1blue (pQEHU) was grown and induced with IPTG as described in Section “Materials and Methods.” Samples were electrophoresed in SDS-12% polyacrylamide gels and polypeptides stained with Coomassie Blue. Left image: expression of (H_6_)SpnHU and purification steps. CE, crude extract (20 μg per lane); 0 and 60 min, samples after IPTG induction; lysate S and lysate P, supernatant and pellet after lysis and centrifugation. Fractions (15 μl) of a Ni-NTA column in which an imidazole gradient was applied. The lane marked with an asterisk contains the (H_6_)SpnHU that was used for further purification. Middle image: DpaseI and Qcyclase treatment followed by Ni-NTA purification. Right image: pGAPase treatment followed by Ni-NTA purification and 3 μg of purified proteins (H_6_)SpnHU and SpnHU. MW, molecular weight markers. **(B)** Oligomerization status of SpnHU. The indicated amounts of crosslinked native protein were separated by SDS-PAGE, and the gels were stained with Coomassie. **(C)** Agarose gel-based EMSAs showing the binding of increasing concentrations of SpnHU to 0.03 pmol supercoiled (s/c), linear (lin) or nicked (nc) plasmid pBR322. **(D)** Agarose gel electrophoresis showing the supercoiling s/c of relaxed (R) pBR322 plasmid after incubation with pneumococcal topoisomerase I in the presence of the indicated amounts of SpnHU (second and consecutive lanes). The first lane shows the DNA molecular weight marker with sizes indicated in kb, and the second lane shows supercoiled pBR322 before relaxation with topoisomerase I.

To assess the *in vitro* activity of SpnHU, the protein was purified to approximately 98% homogeneity (**Figure 2A**) after removal of the H_6_-tag, as described in Section “Materials and Methods.” In solution, the protein formed dimers at all tested concentrations (**Figure 2B**), consistent with the results obtained for other HU proteins (Christodoulou and Vorgias, 2002; Chen et al., 2004; Ghosh and Grove, 2004) and with the cocrystal structures of dimeric Anabaena HU bound to DNA (Swinger et al., 2003). At protein concentrations higher than 10 μM, multimeric species, including trimers, were observed, and some of them failed to enter the gel (**Figure 2B**). The formation of oligomeric structures *in vitro* has also been observed for other HUs and can be explained as the likely association of free monomers, either with themselves or with assembled dimers, resulting in non-specific aggregation (Ghosh and Grove, 2004; Kamau et al., 2005).

The affinity of SpnHU for supercoiled, linear, or nicked DNA was tested by gel-shift assays using 2 nM plasmid pBR322. While shifts in mobility were observed with a concentration of 0.2 μM SpnHU when supercoiled pBR322 was used, eight-fold and sixteen-fold more SpnHU (1.6 μM and 3.2 μM) was required to shift 2 nM linear or nicked pBR322, respectively (**Figure 2C**). This preference for supercoiled DNA has been previously described for other bacterial HU proteins (Chen et al., 2004; Kamau et al., 2005; Mukherjee et al., 2008). SpnHU is also able to efficiently constrain pBR322 relaxed with *S. pneumoniae* topoisomerase I (García et al., 2011), at concentrations lower than 5 nM (**Figure 2D**).

### SpnHU Is Essential for Cell Viability

**FIGURE 3:**
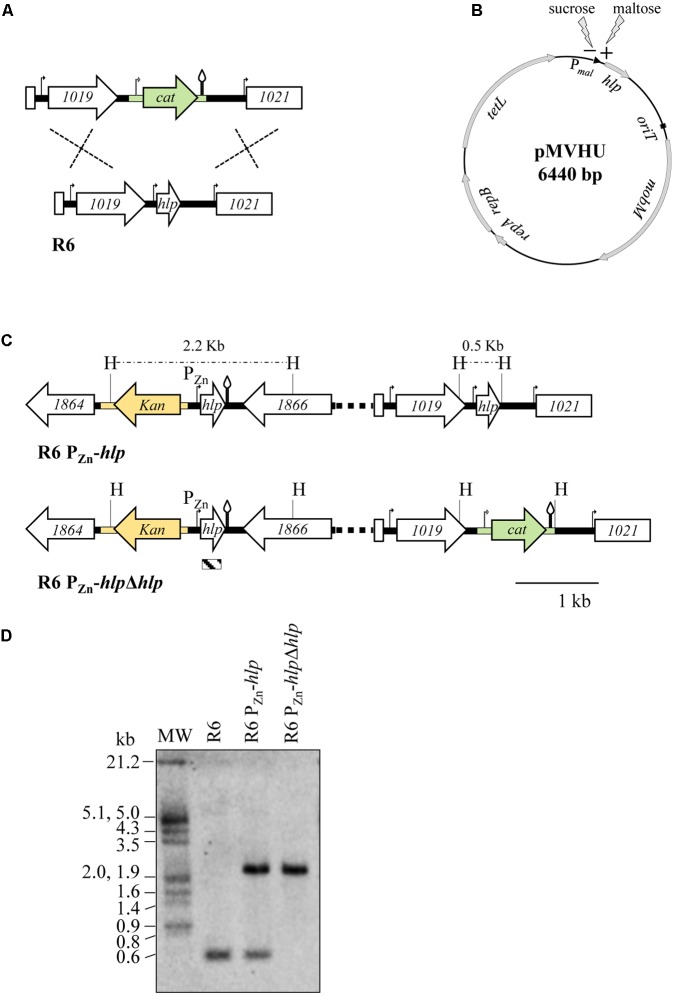
Strategies to knockout the *hlp* gene in *S. pneumoniae.* **(A)** Schematic showing the replacement of the chromosomal *hlp* gene with the chloramphenicol resistance cassette (green drawing). R6 genes (spr numbering) are indicated by white arrows. Promoters are shown as curved arrows and transcription terminators as stem and loop structures. **(B)** Representation of plasmid pMVHU containing the *hlp* gene under the control of the maltose promoter. **(C)** Schematic showing the *in cis* duplication of the *hlp* gene in strain R6 P_Zn_-*hlp*, by placing an additional copy of the gene under the control of a Zn-inducible promoter. The kanamycin resistance cassette is presented in yellow. The organization of strain R6 P_Zn_-*hlp*Δ*hlp* is also represented. The chloramphenicol resistance cassette, promoters, and transcription terminators are represented as in **(A)**, H, HindIII targets and predicted sizes of fragments. Distances between HindIII targets are shown above the dashed lines. The striped box at the bottom of the figure represents the *hlp* probe used for Southern analysis and its position inside the *hlp* gene. **(D)** Southern blot analysis of the R6, R6 P_Zn_-*hlp*, and R6 P_Zn_-*hlp*Δ*hlp* strains. Chromosomal DNA from the strains was cut with HindIII, separated by agarose gel electrophoresis, transferred to a nylon membrane and hybridized with the biotinylated *hlp* probe shown in **(C)**.

Previous studies of other bacteria have shown that most homodimeric HU proteins are essential (Micka and Marahiel, 1992; Liu et al., 2008b), although there are exceptions such as HU of *Mycobacterium smegmatis*, which can be deleted (Hołówka et al., 2017). However, heterodimeric proteins are not essential (Wada et al., 1988). In *S. pneumoniae*, several studies have been conducted to identify essential genes through the use of different high-throughput gene disruption systems and showed discrepancies regarding the essentiality of *hlp* (Thanassi et al., 2002; Song et al., 2005; van Opijnen et al., 2009; van Opijnen and Camilli, 2012; Mobegi et al., 2014; Verhagen et al., 2014). To discern whether SpnHU is essential for cell viability, we attempted to knockout the *hlp* gene by replacing it with a chloramphenicol resistance cassette (*cat*). A fragment of 3.3 kb containing the *hlp* flanking genes *spr1019* and *spr1021* ligated to *cat* was constructed (**Figure 3A**) and introduced into the R6 strain by genetic transformation. The frequency of appearance of chloramphenicol-resistant (Cm^R^) colonies was approximately 2 × 10^4^-fold lower (**Table 2**) than that obtained either by a similar construction generated to replace the dispensable *spxB* gene by *cat* (Ferrándiz and de la Campa, 2014), or by transforming plasmid pJS3 containing *cat* (Ballester et al., 1986). To analyze whether the *cat* gene had been properly integrated, eight Cm^R^ colonies were analyzed by PCR using primers bordering *spr1019* and *spr1021* (**Table 1**). All colonies amplified a fragment of 3.2 kb, consistent with the size of wild-type *hlp* (data not shown). The chloramphenicol resistance of these colonies could be explained by integration of the cassette in other chromosomal loci sharing low homology with homologous arms present in the cassette (the *spr1019* and/ or *spr1020* regions), which would explain the low frequency of transformation. These data suggest that SpnHU is an essential protein. To confirm this finding, the *hlp* gene was either cloned in a plasmid, rendering strain R6 (pMVHU) (**Figure 3B**), or duplicated in the chromosome, rendering strain R6 P_Zn_-*hlp* (**Figure 3C**). In both constructs, *hlp* was cloned under the control of an inducible promoter, such as P*_mal_*, in plasmid pMVHU, which is activated in the presence of maltose (Nieto et al., 2001), and P_Zn_ in strain R6 P_Zn_-*hlp*, which is induced by addition of ZnSO_4_ to the culture (Kloosterman et al., 2007). Strain R6 P_Zn_-*hlp* contained an additional copy of *hlp* ectopically integrated into the *spr1865* locus (**Figure 3C**). The introduction of ectopic DNA in this locus did not affect the cell viability (data not shown). Both strains, R6 (pMVHU) and R6 P_Zn_-*hlp*, were transformed with the 3.3-kb *spr1119*-*cat*-*spr1021*cassette (**Figure 3A**), and colonies were selected under conditions that allowed expression of their ectopic *hlp*. In both cases, Cm^R^ transformants were obtained with transformation frequencies of 2.50% and 1.37% in R6 (pMVHU) and R6 P_Zn_-*hlp* receptor strains, respectively (**Table 2**). These frequencies were in agreement with those obtained with controls: 2.54% for plasmid pJS3 and 1.89% for the 4.5-kb fragment containing *spxB*-*cat* (**Table 2**). Ten Cm^R^ colonies from these transformations of R6 (pMVHU) and R6 P_Zn_-*hlp* were analyzed by PCR using primers bordering *spr1019* and *spr1021* (**Table 1**). All analyzed colonies amplified a 3.9 kb fragment, consistent with the *hlp* replaced with *cat*. The sequence of chromosomal DNA confirmed the deletion of *hlp*. Additionally, strain R6 P_Zn_-*hlp*Δ*hlp* obtained from R6 P_Zn_-*hlp* by deletion of *hlp* (**Figure 3C**) was also checked by Southern blot analysis (**Figure 3D**). All these data support that SpnHU is essential for growth.

**Table 2:** Attempts to disrupt *hlp* in the chromosome of R6 or R6 complemented with *hlp*.

	Recipient strain^b^					
Donor DNA^a^	R6		R6 (pMVHU)		R6 P_Zn_-*hlp*	
	Transformants^c^	Frequency	Transformants	Frequency	Transformants	Frequency
pJS3	1.18 × 10^5^	1.97	1.52 × 10^5^	2.54	–	–
4.5 kb *spxB*-*cat*	1.12 × 10^5^	1.90	ND	ND	1.13 × 10^5^	1.89
3.3 kb *hlp*-*cat*	0.00008 × 10^5^	0.0001	1.50 × 10^5^	2.50	0.82 × 10^5^	1.37

*^a^ pJS3 is a plasmid containing the cat cassette, and the other DNAs are PCR amplification products obtained as described in the text. ^b^ DNA (0.5 μg) was used to transform 1 ml (6 × 10^6^ cells) of competent cultures of R6, R6 (pMVHU) or R6 P_Zn_-hlp strains. Transformants were selected with 2.5 μg/ml chloramphenicol. For R6 (pMVHU), the medium contained 0.8% of maltose as the carbon source, and for R6 P_Zn_-hlp, the medium was supplemented with 0.1 mM ZnSO_4_ for hlp expression. ^c^ Number of transformants per ml. ND, not done. The frequency is provided as a %.*

### The Levels of SpnHU Affect the *in Vivo* Level of DNA Supercoiling

**FIGURE 4:**
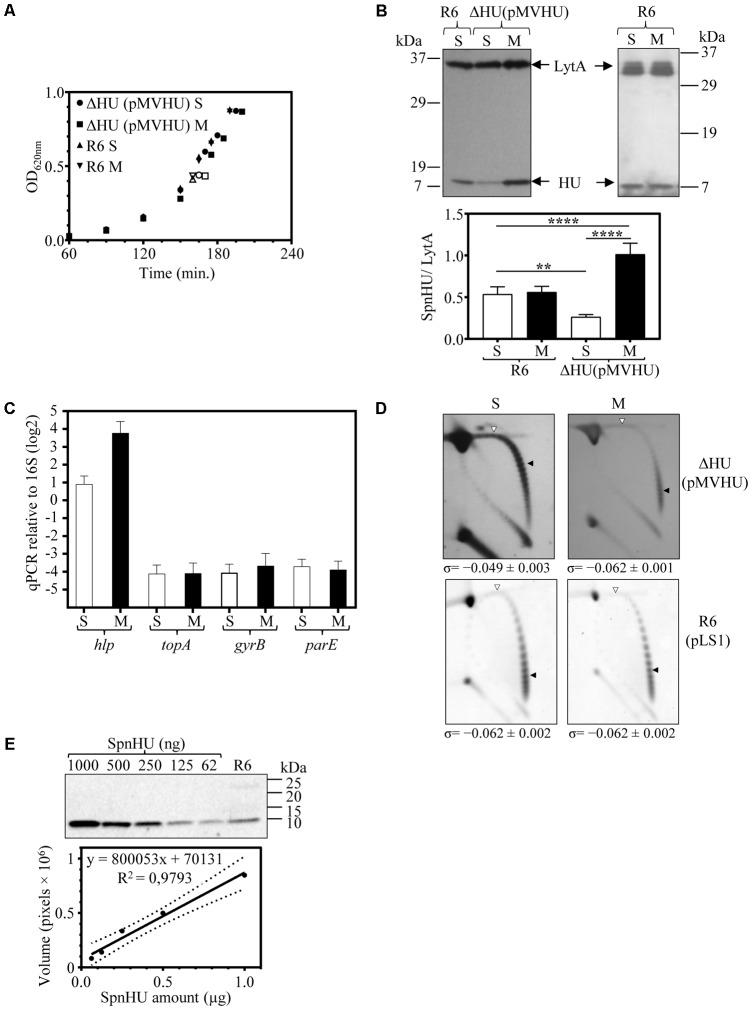
Oscillations in the amount of SpnHU affect the level of DNA supercoiling. Cultures of R6, R6 (pLS1), and ΔHU (pMVHU) were grown in medium containing sucrose. When the cultures reached OD_620__nm_= 0.2, they were diluted 50-fold in medium containing either sucrose (S) or maltose (M). Samples were obtained when the cultures reached OD_620__nm_= 0.4. **(A)** Growth kinetics of ΔHU (pMVHU) and R6 strains grown in sucrose and maltose-supplemented media. Open points indicate the time at which samples were collected. **(B)** Western blot analysis of SpnHU levels in samples grown in medium containing sucrose (S) or maltose (M). Crude cell extracts (20 μg) were separated by SDS-PAGE and blotted. To quantify SpnHU, values were divided by the amount of LytA protein as a loading control. Values are the average ± SD of three independent replicates. (^∗^*P* < 0.0332, ^∗∗∗^*P* < 0.0002). **(C)** qRT-PCR values of *hlp*, *topA*, *gyrB*, and *parE* genes in cells of ΔHU (pMVHU) grown in medium containing maltose or sucrose. Values of represented amplicons were calculated relative to those of 16S rDNA. Represented values are the average ± SD of three independent replicates. **(D)** Plasmid DNAs were isolated and subjected to 2D agarose gel electrophoresis as described in Section “Materials and Methods.” Supercoiling density (σ) values are averages ± SD from three independent replicates. **(E)** Western blot analysis of different amounts of purified SpnHU and a crude cell extract of R6 strain to calculate the number of SpnHU molecules per cell. The linearity of the SpnHU amount and volume expressed is presented as the number of pixels.

To study the influence of SpnHU on DNA supercoiling *in vivo*, we used strain ΔHU (pMVHU). This strain, constructed from R6, contained the chromosomal copy of *hlp* replaced with *cat* and an ectopic copy of the gene cloned under the control of P*_mal_* in plasmid pMVHU (**Figures 3A,B**). As P*_mal_* is inducible by maltose and repressed in the presence of sucrose (Nieto et al., 2001), strain ΔHU (pMVHU) would produce SpnHU, depending on the presence of sucrose or maltose as the carbon source in the growing medium. We grew ΔHU (pMVHU) and its parental strain R6 as a control, either in medium with maltose or sucrose. No remarkable differences in growth were observed among the strains in any of the growth media used (**Figure 4A**). We measured the amount of SpnHU under both conditions in the exponential phase of growth by Western blot analysis. As expected, the amount of SpnHU in R6 did not change in any of the media used (**Figure 4B**). However, for ΔHU (pMVHU), the amount of SpnHU increased significantly by 3.9-fold (*P* < 0.0001) under growth induction conditions (maltose, M) with respect to the values detected under repression conditions (sucrose, S) (**Figure 4B**). Although P*_mal_* was not completely silenced under suppression conditions, the amount of SpnHU decreased significantly by twofold (*P* = 0.0052) compared with R6 grown under the same conditions (**Figure 4B**).

The expression of *hlp* in strain ΔHU (pMVHU) grown in maltose or sucrose was evaluated by qRT-PCR as indicated in Section “Materials and Methods”. The induction of P*_mal_* triggered an increase by 7.3-fold in the expression of *hlp* (**Figure 4C**). Taken together these data revealed that variations in the amount of SpnHU were possible when the P*_mal_* expression system was used in strain ΔHU (pMVHU).

To assess whether these SpnHU variations affected DNA supercoiling levels *in vivo*, the level of chromosome supercoiling in strains ΔHU (pMVHU) and R6 (pLS1) was inferred from the distribution of topoisomers of plasmids pMVHU and pLS1 present in the cells. It has been previously shown that the supercoiling density of small plasmids can be subrogated to the chromosome (Pruss et al., 1982; Weitao et al., 2000; Ferrándiz et al., 2010). Plasmids pMVHU and pLS1 replicate by a rolling circle mechanism (de la Campa et al., 1990), and all their genes are transcribed in the same direction, thus avoiding transcription interference during replication. Plasmids were extracted from cells grown in medium either with sucrose or maltose. The distribution of topoisomers was analyzed by two-dimensional agarose gel electrophoresis in the presence of chloroquine, which intercalates in the DNA and thus permits the separation of molecular DNA species by mass and shape. Chloroquine was used in the second dimension at 2 μg/ml, which induced Δ*Lk* of -20 and -14 for plasmids pMVHU and pLS1, respectively.

Quantification of DNA supercoiling was performed by calculating the supercoiling density (σ) as described in Section “Materials and Methods”. Values of σ for pMVHU extracted from ΔHU cells grown in medium supplemented either with sucrose or maltose were -0.049 ± 0.003 and -0.062 ± 0.001, respectively (**Figure 4D**), which indicated a significant increase of 21% in the value of σ (*P* = 0.0004) when SpnHU levels dropped 3.9-fold. In the case of pLS1 extracted from strain R6 grown in either sucrose or maltose-supplemented medium, the σ values remained the same at -0.062 ± 0.002, (**Figure 4D**), demonstrating that the carbon source employed did not affect DNA supercoiling. Taken together these data revealed that the decrease in DNA supercoiling of ΔHU (pMVHU) grown in sucrose occurred independently of the carbon source and therefore correlated with the decline in the amount of SpnHU. This decrease was 50.9 and 72.3% compared with the R6 grown in either sucrose or maltose, and ΔHU (pMVHU) grown in maltose, respectively (**Figure 4B**). Conversely, we did not observe an increase in the level of negative supercoiling when the levels of protein duplicated, i.e., in the case of ΔHU (pMVHU) grown with maltose compared with R6 grown in either sucrose or maltose (**Figure 4D**).

In pneumococcus, the decrease in negative DNA supercoiling, i.e., relaxation triggers a homeostatic response that involves changes in the expression of genes encoding DNA topoisomerases (Ferrándiz et al., 2010). We examined whether the DNA relaxation caused by the decrease in SpnHU prompted this effect. We measured the expression of genes coding topoisomerase I, gyrase, and one of the subunits of topoisomerase IV: *topA, gyrB*, and *parE*, respectively, by qRT-PCR. No alterations were found in the expression of either *topA, gyrB* or *parE* when ΔHU (pMVHU) was grown in the presence of both sucrose and maltose (**Figure 4C**). This result supports the conclusion that the drop of 50.9% in SpnHU was the responsible for the 21% increase in DNA relaxation, which did not affect the levels of DNA topoisomerases.

To determine the number of SpnHU molecules present in cells during the exponential growth phase, Western blot analysis with different amounts of purified SpnHU and crude extracts of the R6 strain (**Figure 4E**) were performed, as explained in Section “Materials and Methods.” The estimated number of SpnHU molecules was 67240 ± 14700.

### An Increase in SpnHU Attenuated DNA Relaxation Induced by Novobiocin

**FIGURE 5:**
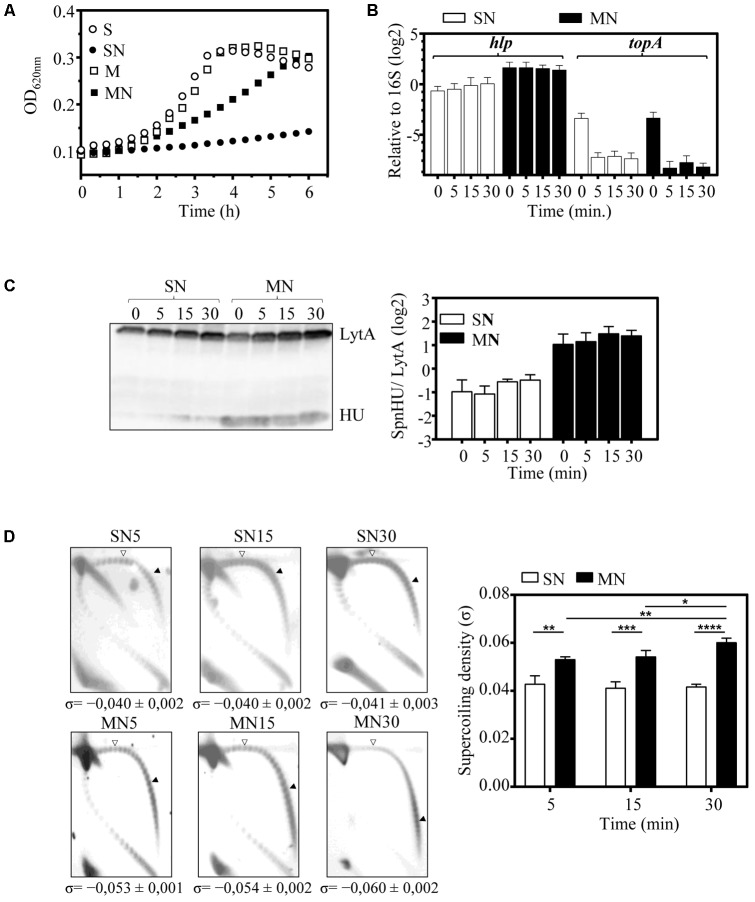
The increase in SpnHU protein levels attenuates the relaxation effect of NOV. A culture of ΔHU (pMVHU) was grown in medium containing sucrose until it reached an OD_620__nm_= 0.2, and then it was diluted 50-fold in four different media: two containing either sucrose (S), or maltose (M) and two containing either sucrose or maltose, with 1 μg/ml of NOV (SN or MN). Cells were collected 5, 15, and 30 min after addition of the drug. **(A)** Growth kinetics of ΔHU (pMVHU) cultures. **(B)** qRT-PCR values of *hlp* and *topA* genes relative to those of 16S rDNA after treatment with NOV. Represented values are the average ± SD of three independent replicates. **(C)** Western blot analysis of SpnHU protein levels in cells exposed to NOV. Crude cell extracts (20 μg) were separated by SDS-PAGE, blotted, and incubated with antibodies directed against SpnHU and LytA. To quantify SpnHU, values were divided by the amount of LytA protein as a loading control. Three independent replicates are represented. **(D)** Plasmid DNA from cells exposed to NOV at different times was isolated and subjected to 2D agarose gel electrophoresis as described in Section “Materials and Methods.” Supercoiling density (σ) values are averages ± SD from three independent replicates. (^∗^*P* < 0.0332, ^∗∗^*P* < 0.0021, ^∗∗∗^*P* < 0.0002, ^∗∗∗∗^*P* < 0.0001).

To further analyze the contribution of SpnHU to DNA supercoiling levels, ΔHU (pMVHU) was grown in medium containing either sucrose or maltose in the absence or presence of novobiocin (NOV), which specifically inhibits the activity of gyrase B subunit, leading to DNA relaxation (Ferrándiz et al., 2010). No differences in growth were found when ΔHU (pMVHU) was cultured with sucrose or maltose in the absence of NOV (duplication times of 84.1 ± 2.1 min and 84.1 ± 4.3 min, *n* = 3, respectively) (**Figure 5A**). This finding shows that small changes in SpnHU protein levels do not contribute to a loss in cell viability. However, in the presence of 1 μg/ml NOV (1 × MIC), ΔHU (pMVHU) only grew in the maltose medium (doubling time of 288.5 ± 26.4 min, *n* = 3) (**Figure 5A**), which shows that more SpnHU partially counteracts the effect of NOV on DNA supercoiling.

It has been previously shown that the treatment of *S. pneumoniae* with NOV induces DNA relaxation and triggers a homeostatic response to restore the level of DNA supercoiling when the induced relaxation is less than 25% (Ferrándiz et al., 2010). This response includes transcriptional changes in topoisomerase genes: up-regulation of gyrase genes and down-regulation of topoisomerase I and IV genes (Ferrándiz et al., 2010). Among topoisomerases, topoisomerase I (encoded by *topA*) is mainly responsible for the maintenance of DNA supercoiling levels in *S. pneumoniae*, since the amount of *topA* mRNA correlated with the level of DNA supercoiling during cellular homeostatic responses either to DNA relaxation or to increased negative DNA supercoiling (Ferrándiz et al., 2016). Expression of both *topA* and *hlp* genes was measured by qRT-PCR after treatment of ΔHU (pMVHU) with NOV. We observed decreases of 16- and 24-fold (mean of 5, 15, and 30 min) in *topA* mRNA when the medium contained sucrose and maltose, respectively (**Figure 5B**). These levels were in the range of those observed previously (Ferrándiz et al., 2010), and indicated that NOV was working as expected. For *hlp*, 3.6-fold (mean of 5, 15, and 30 min) overexpression was detected in cells grown with maltose compared with cells grown with sucrose (**Figure 5B**). This overexpression of *hlp* corresponded to a fourfold (mean of 5, 15, and 30 min) increase in the amount of SpnHU (**Figure 5C**). The difference in the amount of SpnHU observed between cells grown with maltose and cells grown with sucrose remained similar in the presence and absence of NOV in ΔHU (pMVHU) (**Figures 4B**, **5C**). This result is expected since the *hlp* gene in this strain is under the control of the inducible promoter of maltose, the activity of which is not affected by NOV. Based on these results, we deduced that more SpnHU would allow the survival of ΔHU (pMVHU) to 1 × MIC NOV treatment probably because the level of DNA supercoiling was closer to the physiological value.

The DNA supercoiling level of ΔHU (pMVHU) grown with sucrose and maltose and in the presence or absence of 1 × MIC NOV was analyzed by two-dimensional agarose gel electrophoresis. Treatment of ΔHU (pMVHU) grown in sucrose with NOV resulted in σ = -0.040 (mean of 5, 15, and 30 min) (**Figure 5D**). This value was 18.4% (*P* = 0.03) and 35.5% (*P* = 0.0001) lower than those of ΔHU (pMVHU) grown in sucrose and without NOV (σ = -0.049) and R6 grown both in sucrose or maltose and without NOV (σ = -0.062), respectively (**Figure 4D**). The 35.5% drop in the level of DNA supercoiling in ΔHU (pMVHU) grown in sucrose with NOV resulted from the sum of the relaxation induced by both the decrease in the amount of SpnHU (**Figures 4B**,**D**) and NOV treatment (**Figure 5D**). This DNA supercoiling level could not be restored over time, as observed for R6 treated with concentrations of NOV that induced decreases in supercoiling higher than 25% (Ferrándiz et al., 2010). When we compared the DNA supercoiling of ΔHU (pMVHU) grown in maltose with NOV, we observed a significant decrease of 14.5% in DNA supercoiling 5 min after NOV addition with respect to the untreated ΔHU (pMVHU) or R6 (*P* = 0.0002 and *P* = 0.0005, respectively) (**Figures 4D**, **5D**). This decrease was 1.7-fold lower than that observed for R6 treated with the same concentration of NOV (Ferrándiz et al., 2010) and correlated with a 2.1-fold higher amount of SpnHU (**Figure 4B**). The level of DNA supercoiling was fully recovered after 30 min of treatment (**Figure 5D**).

## Discussion

We investigated SpnHU in this study, first by characterizing its biochemical activity and second by analyzing its activity *in vivo*. In terms of its *in vitro* activity, we have observed that it behaves like most of their homologous HU proteins. It preferably forms dimers and binds to supercoiled DNA with higher affinity than to linear or nicked DNA. Furthermore, it is able to constrain relaxed DNA in the presence of topoisomerase I in an efficient manner.

Regarding the *in vivo* activity of SpnHU, we have shown that this protein is involved in the maintenance of DNA supercoiling, and only small variations in the amount of SpnHU are possible to avoid disturbing the proper DNA topology. Therefore, a tight regulation of *hlp* would be expected to preserve an adequate amount of this protein. Transcriptional regulation of *hlp* in *S. pneumoniae* would differ from that of the *hupA* and *hupB* genes in *E. coli*, the expression of which is modulated by Fis and by the catabolite repressor protein CRP (Claret and Rouvière-Yaniv, 1996). Fis is absent in *S. pneumoniae*, and *hlp* is most likely regulated by global changes in DNA supercoiling. Moreover, this gene is located in one of the topology-reactive gene clusters, or domains, in which the pneumococcus genome is divided (Ferrándiz et al., 2010). Genes in these domains show a coordinated transcriptional regulation in response to topological changes (Ferrándiz et al., 2010; de la Campa et al., 2017). Specifically, *hlp* is located in a domain in which transcription is down-regulated when the DNA is relaxed by the inhibition of DNA gyrase by NOV. This kind of regulation by DNA supercoiling also affects all the DNA topoisomerase genes in *S. pneumoniae* (Ferrándiz et al., 2010). Transcriptional down-regulation of *hlp* would be involved in the known homeostatic response to DNA relaxation by which gyrase genes *gyrA* and *gyrB* are up-regulated and *topA* is down-regulated (Ferrándiz et al., 2010). All these changes would balance the DNA supercoiling after relaxation induced by NOV.

In addition, we have shown that SpnHU is essential for the viability of pneumococcal R6 cells, supporting some previous studies of essential genes in *S. pneumoniae* (Thanassi et al., 2002; Song et al., 2005; Mobegi et al., 2014). These studies were performed with strain D39, its derivatives Rx1 and R6, and TIGR4. Other studies in which *hlp* was not essential, used TIGR4, D39, and the Spain^9V^-3 SP195 strain (van Opijnen et al., 2009; van Opijnen and Camilli, 2012; Verhagen et al., 2014). Differences with respect to the essentiality of the *hlp* gene may be related to the length of the gene, with short genes such as *hlp* (273 bp) being less susceptible to disruption by transposon insertions. In fact, among all the essential genes found in two of the mentioned studies (van Opijnen et al., 2009; Mobegi et al., 2014), only 9% have sizes < 300 bp despite representing 18% of the genome. However, 81% have sizes > 300 bp (representing 91% of the genome).

The survival of *S. pneumoniae* seems to be possible only when at least a minimum amount of this protein is present in the cell. The cellular dependence of this protein could be due to its importance in preserving DNA topology, which is crucial for DNA metabolic processes. In fact, in *E. coli*, HU modulates the transcription of genes that respond to stress conditions and SOS induction (Oberto et al., 2009), and a possible role in the coordination of replication with chromosome segregation has also been observed in *Mycobacterium tuberculosis* as HupB in this bacterium preferably associates with the origin organizing the newly replicated *oriC* regions (Hołówka et al., 2017). In the phylogenetically related *S. intermedius*, SiHU downregulation leads to an alteration of the cell transcription program, with changes in nucleoid segregation, cell division and cell surface properties (Liu et al., 2008b).

In contrast to other bacteria, such as *E. coli*, the landscape of NAPs in *S. pneumoniae* is drastically reduced such that NAPs such as FIS, H-NS, or IHF are not present. The same occurs in other bacteria of the Firmicutes phylum, such as *B. subtilis*, which also lacks FIS, H-NS, or IHF. However, this bacterium has some DNA-bridging proteins that belong to the Lrp family, which could be involved in the maintenance of DNA topology (Beloin et al., 1997). These proteins are absent in *S. pneumoniae*, although we cannot exclude the possibility that a structural homologue of Fis or other NAPs are present. This situation suggests that SpnHU plays an important role in the preservation of DNA supercoiling. Indeed, the observed 21% reduction of the supercoiling level when the cell has a twofold lower amount of SpnHU can be ascribed to the wrapping property of this protein since the level of topoisomerases, which are mainly responsible for supercoiling variations, does not change. In *S. intermedius*, downregulation of SiHU is possible due to the induced expression of a Si-*hlp* antisense RNA, which triggers nucleoids expansion (Liu et al., 2008b), supporting the decrease in HU and leading to a lower level of restrained DNA supercoils.

Taken together these results show that *in vivo*, SpnHU contributes to the maintenance of DNA supercoiling and that this essential protein can only be partially removed. The reduction of SpnHU seems to only be possible up to a level where the negative supercoiling is not reduced beyond 20%, which is the limit of cell survival (Ferrándiz et al., 2010). Although up to a 40% increase in negative supercoiling can be tolerated by the cell (Ferrándiz et al., 2016), hypernegative supercoiling is not observed when SpnHU levels are increased. Similarly, overproduction of HU from *E. coli* causes no change in DNA supercoiling (Mc Govern et al., 1994). Unlike *E. coli*, in which the protein is induced approximately 40-fold, a moderate increase (only fourfold) was observed herein. The possible effect of a saturation of SpnHU bound to the DNA does not explain the inability of the increase in HU to result in an increase in supercoiling. In fact, HU and Fis are the most abundant NAPs in exponentially growing *E. coli* cells. The approximate number of dimeric molecules per cell is 30000 (Ali Azam et al., 1999), which would allow HU dimers to be uniformly distributed along the *E. coli* chromosome every ≈ 190 bp (Azam et al., 2000). In *S. pneumoniae*, we estimated that the number of SpnHU molecules per cell is approximately 34000 dimers (67240 ± 14700 monomers), which is in the same range as that observed for *E. coli*. However, the size of pneumococcus chromosome (2038615 bp) is almost half that of *E. coli*, resulting in an HU distribution of 1 dimer per ≈ 60 bp. As in *E. coli*, the amount of SpnHU is not enough to coat the entire chromosome, taking into account that HU dimers need 21 bp DNA to form a helical complex, as demonstrated for *Anabaena* HU-DNA cocrystals (Swinger et al., 2003). The formation of flexible bends induced by HU dimers has been proposed to explain the DNA compaction. These nucleosome-like structures are rescinded in the case of high HU to DNA ratios due to the formation of a rigid nucleoprotein filament (Sagi et al., 2004; van Noort et al., 2004) potentially explaining why increases in HU not result in hypernegative supercoiling.

We found that a moderate increase in SpnHU levels attenuates the effect of relaxation induced by NOV, in agreement with our biochemical results. SpnHU constrains the supercoiling, counteracting the effect of a less active gyrase. Both a drop in topoisomerase I and an increased amount of SpnHU can attenuate the effect of the drug, restoring an adequate DNA supercoiling balance and thus allowing cells to grow at the inhibitory concentration of NOV. Taken together, these data seem to indicate that the amount of this protein is critical for pneumococcus survival, and ability to cope with moderate changes in DNA supercoiling.

## Author Contributions

AC and M-JF conceived, designed, and supervised the study. DC purified (H_6_)SpnHU and participated with SA in the EMSA experiments. M-JF carried out the remaining experiments and wrote the manuscript. All authors read and actively participated in the correction of the manuscript. The manuscript has been approved by all authors for publication.

## Conflict of Interest Statement

The authors declare that the research was conducted in the absence of any commercial or financial relationships that could be construed as a potential conflict of interest.
